# The Hippo Pathway: Immunity and Cancer

**DOI:** 10.3390/cancers10040094

**Published:** 2018-03-28

**Authors:** Zaid Taha, Helena J. Janse van Rensburg, Xiaolong Yang

**Affiliations:** Department of Pathology and Molecular Medicine, Queen’s University, Kingston, ON K7L 3N6, Canada; zaid.taha@queensu.ca (Z.T.); 8vrhjj@queensu.ca (H.J.J.v.R.)

**Keywords:** Hippo pathway, cancer, immunology, immunotherapy, inflammation, MST1/2, LATS1/2, YAP, TAZ, PD-L1

## Abstract

Since its discovery, the Hippo pathway has emerged as a central signaling network in mammalian cells. Canonical signaling through the Hippo pathway core components (MST1/2, LATS1/2, YAP and TAZ) is important for development and tissue homeostasis while aberrant signaling through the Hippo pathway has been implicated in multiple pathologies, including cancer. Recent studies have uncovered new roles for the Hippo pathway in immunology. In this review, we summarize the mechanisms by which Hippo signaling in pathogen-infected or neoplastic cells affects the activities of immune cells that respond to these threats. We further discuss how Hippo signaling functions as part of an immune response. Finally, we review how immune cell-intrinsic Hippo signaling modulates the development/function of leukocytes and propose directions for future work.

## 1. Introduction

### 1.1. Signaling Pathways in Immunology

The immune system plays important roles in health and disease. Through various cell types and a multitude of secreted factors, the immune system defends the human body from internal and external threats. While the interactions of the immune system are complex, from a reductionist perspective, immune responses can be conceptualized as a series of signaling events occurring within and between immune cells, healthy cells, pathogen-infected cells and/or neoplastic cells. Indeed, specific signaling pathways (including NF-κB, Toll-like receptor (TLR), interferon (IFN) and JAK/STAT pathways) have been found to coordinate many of the intricacies of an immune response whereas dysregulated signaling resulting in altered immune function has been shown to contribute to disease pathology [1,2,3,4]. Given this, it is vital that we understand how particular signaling networks regulate immune processes in order to decipher mechanisms of disease and to identify opportunities for therapeutic intervention.

### 1.2. Hippo Signaling in Drosophila and Mammals

**Figure 1:**
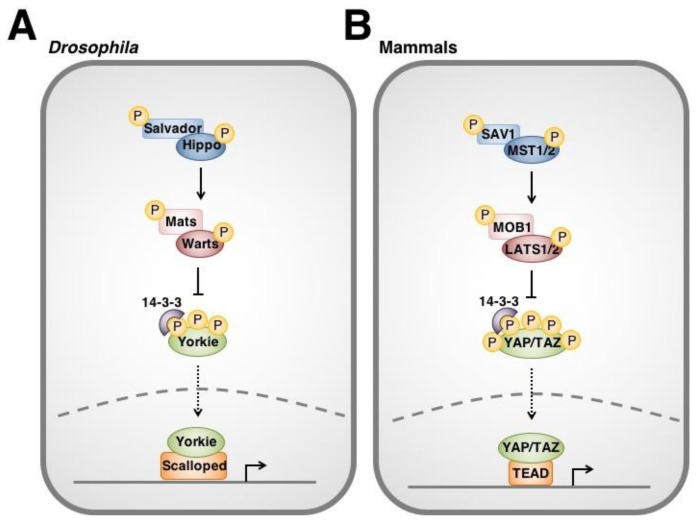
Overview of the Hippo signaling pathway in *Drosophila* (**A**) and mammals (**B**). Hippo signaling is initiated by a variety of upstream stimuli. Activation of Hippo (MST1/2) leads to subsequent phosphorylation of Warts (LATS1/2). Warts negatively regulates the Hippo pathway effector Yorkie (YAP/TAZ). Unphosphorylated Yorkie translocates into the nucleus where it interacts with its Scalloped (TEAD) transcription factors to upregulate the transcription of a variety of genes. In contrast, phosphorylation of Yorkie by Wts lead to its cytoplasmic sequestration by 14-3-3 proteins and degradation.

The Hippo signaling pathway was originally discovered through a series of genetic mosaic screens for genes augmenting cell proliferation and organ size in *Drosophila* [5,6,7,8,9,10,11,12]. In canonical Hippo signaling (Figure 1A), upstream stimuli activate the Hippo (Hpo) serine/threonine kinase. Hpo forms a complex with Salvador (Sav) scaffold protein and Mob as a tumour suppressor (Mats) adaptor protein to phosphorylate and activate serine/threonine kinase Warts (Wts aka. large tumour suppressor, lats) [8,9,10,11]. Wts subsequently phosphorylates transcriptional co-activator Yorkie (Yki) at key serine residues [13,14,15]. Phosphorylation of Yki by Wts leads to sequestration of Yki by 14-3-3 proteins in the cytoplasm. Thus, Yki is prevented from entering the nucleus to interact with Scalloped transcription factors, cannot trans-activate gene targets (e.g., *Drosophila* inhibitor of apoptosis protein 1 (*Diap1*)) and is functionally inhibited by Hippo signaling [13].

Since its discovery in *Drosophila*, homologs for each component of the Hippo pathway have been identified in other species. In mammals (Figure 1B), mammalian sterile 20-like kinase 1/2 (MST1/2) kinases associate with Salvador family WW domain containing protein 1 (SAV1) and Mps one binder kinase activator-like 1A and 1B (MOB1A/B or collectively, MOB1) to phosphorylate large tumour suppressor 1/2 (LATS1/2). LATS1/2 subsequently phosphorylate Yes-associated protein (YAP) as well as its paralog, WW domain-containing transcription regulator 1 (TAZ), leading to their binding by 14-3-3 [16,17,18,19]. YAP and TAZ are thereby prevented from entering the nucleus, interacting with transcription factors (i.e., TEAD family members and others) and regulating downstream gene targets [20,21,22,23,24].

The last decade has seen great advances in our understanding of the Hippo pathway. A diverse range of regulatory factors/cellular processes that influence MST1/2 and LATS1/2 activity have been uncovered including G protein-coupled receptor (GPCR) signaling, receptor tyrosine kinase (RTK) signaling, cell-cell contact and actin dynamics [25,26]. Non-canonical (“Hippo-independent”) interactions have been described for various components of Hippo signaling [27,28,29,30]. Finally, screens for downstream gene targets regulated by YAP and TAZ (e.g., *CTGF* and *CYR61*) have provided new insights into physiological/pathological functions of the Hippo pathway effectors [31,32,33].

A relatively recent development in the Hippo pathway field is literature investigating interactions between Hippo signaling and the immune system. In this review, we summarize the current evidence demonstrating a relationship between the Hippo pathway and immunology. We describe the mechanisms by which Hippo signaling in pathogen-infected cells regulates the recruitment and behaviour of the immune cells that respond to these pathologies. We further examine the emerging data linking dysregulated Hippo pathway activity in neoplastic cells with cancer immune evasion. We explain how Hippo signaling makes up part of an immune response. We then turn our attention towards leukocytes and highlight how immune cell-intrinsic Hippo signaling is crucial in normal immune cell function. Finally, we propose opportunities for future work.

## 2. Hippo Signaling Regulates Immune Cell Recruitment and Activation

### 2.1. Hippo Signaling Modulates the Tumour Microenvironment in Cancer

Immune cells comprise an important group of non-neoplastic cells that exist within a tumour [34,35]. Tumour-infiltrating immune cells can play a critical role in determining the fate of a neoplastic lesion and can impose selective pressures on evolving cancers. Conversely, cancer cells can manipulate immune cell function to take advantage of tumour-promoting effects while escaping antagonistic activities.

There is compelling evidence that YAP-induced cytokine expression has functional significance in immune-related phenomenon including cancer. Indeed, within the last two years it has become evident that cytokine upregulation by YAP can modify the tumour microenvironment. Recruitment of immunosuppressive cell types (e.g., myeloid-derived suppressor cells (MDSCs)) into a neoplasm represents one mechanism by which YAP establishes a niche for cancer growth. In 2016, Wang et al. used a murine prostate adenocarcinoma model to demonstrate that YAP-driven CXCL5 production by cancer cells can lead MDSCs to the tumour site through heterotypic CXCL5 binding to CXCR2 receptors [36]. Inhibition of the CXCL5-CXCR2 axis or MDSC depletion in this model enhanced anti-tumour immune responses. These findings may have translational relevance for human cancer, as the authors of this study showed that YAP1 activation is associated with an MDSC gene expression signature in prostate cancer clinical datasets. Murakami et al. subsequently reported similar observations in a mouse model of pancreatic ductal adenocarcinoma (PDAC) as well as in clinical datasets from human PDAC [37]. YAP has further been found to function downstream of the PRKCI oncogene to upregulate TNFα expression, recruit MDSCs and inhibit cytotoxic T cell (CTL) function in a mouse model of high-grade serous ovarian carcinoma [38].

**Figure 2:**
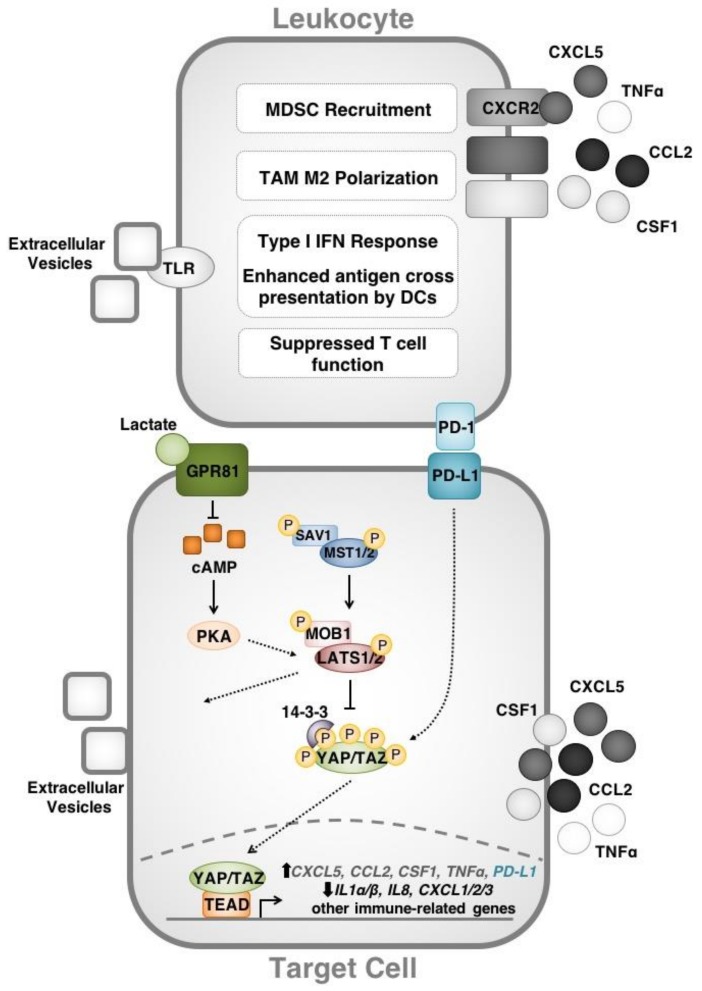
Overview of the Hippo signaling pathway in modifying the anti-neoplastic immune response. The Hippo pathway effectors YAP/TAZ regulate gene targets that direct immune cell function. Cytokines upregulated by YAP including CXCL5 and TNFα recruit myeloid-derived suppressor cells (MSDCs) to the tumour microenvironment while others (CSF1 and CCL2) direct tumour-associated macrophage (TAM) M2 polarization. YAP/TAZ act downstream of Hippo signaling (as well as GPCR signaling) to directly enhance PD-L1 expression and disrupt T cell function through PD-L1/PD-1 binding. Finally, LATS, YAP and TAZ affect the nucleic acid content of extracellular vesicles that modify Toll-like receptor (TLR) signaling, the type 1 interferon (IFN) response and antigen presentation by dendritic cells (DCs). Interactions that have been described in human cells are shown in colour while those that have been demonstrated in murine cell lines are shown in greyscale.

An additional mechanism by which YAP modulates the tumour microenvironment is through interactions with macrophages. Indeed, YAP has been reported to guide the polarization of tumour-associated macrophages (TAMs) towards an immunosuppressive/“pro-tumour” M2 phenotype. Guo and coauthors showed that single tumour-initiating cells can recruit M2 macrophages through YAP-induced expression of CCL2 and CSF1 in a mouse model of liver tumourigenesis [39,40]. In this system, the tumour-associated macrophages recruited by YAP were essential for immune evasion and tumourigenesis. YAP has likewise been implicated in M2 TAM polarization by colon cancer cells [41]. When co-cultured with human colon cancer cell lines (HCT116 or DLD-1), THP-1 monocytic cells differentiate towards an M2 phenotype. YAP knockdown in cancer cells suppresses the M2 differentiation phenotype. Collectively, these studies demonstrate that YAP directs myeloid cell recruitment and behaviour towards functions that enhance tumourigenesis. Therefore, by determining the immune cell content of tumours, the Hippo pathway may play an even greater role in tumourigenesis and cancer progression than was previously appreciated (Figure 2).

### 2.2. The Hippo Pathway Regulates Adaptive Immune Responses

Apart from directing the innate immune cell composition of the tumour microenvironment, Hippo pathway proteins have also been demonstrated to influence adaptive immune responses in multiple disease contexts. For example, Ramjee et al. (2017) have linked the expression of epicardial YAP/TAZ to improved recovery following myocardial infarction (MI) characterized by reduced deleterious cardiac remodeling [42]. The protective effects of YAP/TAZ are thought to be linked to their immunosuppressive effects through IFNγ signaling and recruitment of T_reg_ cells, both of which localize and limit the cardiac inflammatory response. YAP activation through TLR3 signaling has similarly been reported to enhance neonatal cardiac functional recovery following MI [43]. The anti-inflammatory effects of YAP/TAZ reported in these studies are consistent with the pro-inflammatory effect of MST1 in cerebral acute ischemia-reperfusion injury that was described by Zhao et al. (2016) [44]. In this model, MST1 mediates neuronal cell death through NFκB-induced microglial activation following cerebral infarction.

A number of recent reports have demonstrated a direct link between Hippo signaling and suppression of CD8+ and CD4+ T cell function in the context of cancer. We and others have recently observed that the Hippo pathway effectors YAP and TAZ directly upregulate the expression of the immune checkpoint molecule programmed death ligand-1 (PD-L1), thereby suppressing anti-neoplastic T cell responses in a number of different in vitro models. Specifically, we reported a correlation between TAZ and PD-L1 protein levels in human breast and lung cancer cell lines [45]. Through ChIP and luciferase assays, we showed that the TAZ/YAP/TEAD4 complex enhances *PD-L1* promoter activity and we demonstrated that the relationship between TAZ and PD-L1 has functional significance in cancer immune evasion through co-culture experiments. Additionally, we determined that upstream regulators (e.g., insulin, S1P, PI3K, RAF) and components (i.e., MST1/2, LATS1/2) of the Hippo pathway also regulate PD-L1 expression, suggesting that Hippo signaling may contribute to immune evasion through PD-L1. Interestingly, we were not able to reproduce the relationship between YAP/TAZ and PD-L1 in murine cell lines, suggesting that this regulatory mechanism may not be conserved in mouse models. Lee et al. (2017) characterized YAP as a transcriptional regulator of PD-L1 in human lung adenocarcinoma cells [46]. In their report, the authors described a connection between epidermal growth factor receptor (EGFR) tyrosine kinase inhibitor (TKI) resistance in lung adenocarcinoma cells with both YAP and PD-L1 expression. YAP knockdown conferred a significant reduction in PD-L1 levels. These results were very recently reproduced by other groups in non-small cell lung cancer (NSCLC) and BRAF-inhibitor resistant melanoma cell lines, reinforcing the role of the Hippo effector YAP in the transcriptional regulation of PD-L1 expression [47,48].

The involvement of Hippo signaling in mediating localized immunosuppression through PD-L1 is a critical mechanism by which Hippo pathway proteins reprogram the tumour micro-environment. In this case, YAP/TAZ modulate the tumour-immune cell interface by dampening adaptive T cell responses. This finding may provide new insights into stimuli that can regulate PD-L1 expression and cancer immune evasion through modifying Hippo signaling. For example, in their work on TAZ-dependent PD-L1 upregulation, Feng et al. demonstrated that Hippo signaling in human lung adenocarcinoma is affected by the pH of the extracellular environment, and this in turn leads to TAZ-mediated upregulation of PD-L1 [49]. More specifically, Feng et al. described a correlation between tumour lactate levels and PD-L1 expression. In this model, G-protein coupled receptor 81 (GPR81) initiates lactate-induced PD-L1 upregulation through depletion of intracellular cAMP levels, inhibition of protein kinase A (PKA) and activation of TAZ. Therefore, the Hippo network may link physical/chemical/biological stimuli with immunosuppressive reprogramming of the tumour microenvironment.

Reports of cancer cell-intrinsic functions for PD-L1 have added an additional layer of complexity to the relationship between the Hippo pathway and PD-L1. Several groups have proposed that PD-L1 can signal within cancer cells to escape cytotoxicity and also to promote chemotherapy resistance and metastasis [50,51]. Surprisingly, PD-L1 may be responsible for upregulating YAP expression levels in NSCLC lines. Tung et al. recently reported that PD-L1 expression in NSCLC lines was associated with increased generation of reactive oxygen species (ROS), which leads to upregulation of hypoxia inducible factor 1α (HIF1α) [52]. As a result, PD-L1 overexpression effectively increases YAP levels and transcriptional activity as well as YAP-induced TKI drug resistance in this NSCLC model. Thus, Hippo signaling may exist as part of a feedback system in which YAP/TAZ-induced PD-L1 expression may further increase the activity of these Hippo pathway effectors.

While most evidence points towards an immunosuppressive function for YAP/TAZ in cancer and other pathologies, there is some conflicting data that remains to be reconciled. Although counter-intuitive, recent findings by Moroishi et al. suggest that LATS1/2 contribute towards the generation of an immunosuppressive tumour microenvironment in vivo [53]. *LATS1/2* double knockout (DKO) (or YAP/TAZ-overexpressing) mouse cancer cells were shown to be highly tumorigenic in vitro, while proving to be poorly tumorigenic in vivo in immunocompetent mice, compared to their wildtype parental controls. Moroishi and colleagues demonstrated that the protective effects of *LATS1/2* DKO stems from the ability of these cells to secrete large amounts of extracellular vesicles abundant in nucleic acids. This cargo is detected by TLRs that induce a type I IFN response, which stimulates adaptive immunity through increased dendritic cell (DC) maturation/antigen cross-presentation as well as increased CTL clonal expansion. Based on these observations, the authors suggest that controlled targeting of LATS1/2 may prove therapeutically efficacious in enhancing tumour immunogenicity in B16-OVA melanoma, 4T1 breast cancer, and SCC7 squamous cell carcinoma mouse models. However, it is unclear how these observations can be squared with the immunosuppressive roles for YAP/TAZ established in other studies. One possibility is that there are species-specific differences in YAP/TAZ transcriptional targets that can account for these divergent observations. Indeed, in our recent characterization of immune-related transcriptional targets of TAZ, we performed NanoString screens using both TAZ-overexpressing human and mouse cell lines [45]. We found many gene targets that appeared to be differentially regulated by TAZ between the two species including *PD-L1*. Given this, we suggest that the relationship between the Hippo pathway and immune cells in human cancers may not be fully recapitulated in mouse models. Indeed, it will be interesting for future studies to compare Hippo pathway functioning across species.

## 3. Hippo Signaling Is Part of an Immune Response

### 3.1. Pathogenic Immune Challenges Regulate Hippo Pathway Proteins

A wide variety of upstream regulators of Hippo signaling exist and include a number of RTKs and GPCRs. TLRs—critical molecules in the primary innate immune response against conserved microbial signatures—have also been found to act as upstream regulators of the Hippo pathway. Using *Drosophila* models, Liu et al. demonstrated that Hippo signaling mediates innate immune response within the *Drosophila* larval fat body immune organ [54]. Liu et al. showed that Yki leads to the suppression of NFκB family transcriptional factors, Dorsal (D1) and Dorsal-related immune factor (Dif), ultimately inhibiting an antimicrobial response. Immune challenge by Gram-positive bacteria led to activation of TLRs, which act through MyD88 and Pelle to further activate Hippo signaling and phosphorylate Yki, thereby stimulating the transcription of antimicrobial proteins by D1 and Dif.

Yki has also been implicated in the transcription of midgut antimicrobial cytokine Unpaired 3 (Upd3), in *Drosophila* enterocytes and enteroblasts, following infection by strains of *Pseudomonas* and *Erwinia carotovora* [55]. While Upd3 induces a number of downstream pathways involved in innate immunity such as Jak/Stat signaling, its primary antimicrobial function in this system is to drive rapid cellular turnover leading to bacterial clearance. This mechanism is consistent with the canonical proliferative functions of Yki/YAP signaling and highlights another way in which the Hippo pathway might contribute to anti-pathogenic immunity. These findings have been similarly described in mice, in the context of helminth infection [56]. Mice with targeted disruption of SET domain-containing protein 7 (*Setd7*) showed increased resistance to intestinal infection of helminthic origin by *Trichuris muris*. In the absence of SETD7, intestinal epithelial cells exhibited increased YAP signaling, increased proliferation, and increased turnover, leading to clearance of parasite load. Collectively, these findings suggest that in addition to directly modulating immune signaling, the Hippo pathway may alter cell susceptibility to infection through reprogramming of proliferation kinetics.

Although TLRs have been shown to upregulate the production of antimicrobial proteins through canonical Hippo signaling, non-canonical functions for MST1/2 have been reported in the context of *Mycobacterium tuberculosis* (*Mtb*) pathogenesis. Boro et al. (2016) showed that *Mtb* infection in murine cells triggers a TLR2 signaling cascade through interleukin receptor-1 associated kinases 1/4 (IRAK1/4) which, in turn, activate MST1/2 [57]. MST1/2 then activates IRF3 to stimulate production of CXCL1/2 and antimicrobial peptides (e.g., β-defensin). These findings suggest that different pathogen-specific roles for Hippo signaling may exist.

In terms of innate antiviral immunity, YAP has been reported to negatively regulate the type I IFN response through inhibition of IRF3 transcriptional activity in mouse and human cells [58]. This mechanism represents a non-canonical function for YAP, mediated through interactions between Inhibitor of nuclear factor kappa B kinase subunit epsilon (IKKε) and two isoforms of YAP (YAP2/4). In the presence of vesicular stomatitis virus (VSV), Sendai virus (SeV) or herpes simplex virus-1 (HSV-1) infection, IKKε phosphorylates YAP2/4 at a LATS1/2-independent site, Ser403, leading to YAP degradation. This phosphorylation relieves YAP-mediated inhibition of the cellular antiviral response by enabling dimerization and nuclear translocation of IRF3. Although this effect was concluded to be mediated solely through IKKε, there was no examination of MST1/2 role in this process. Considering the notable involvement of MST1/2 in the *Mtb*-triggered immune response, it is reasonable to suggest that the role of these kinases in innate antiviral immunity warrants further investigation.

While it is clear that immune challenges modulate Hippo signaling to effect an immune response, there are also mechanisms by which immune challenges can exploit Hippo signaling to enhance pathogenicity. For example, Meng et al. (2016) have reported that the innate cellular antiviral defenses against VSV, SeV and HSV-1 infections involve IRF3 and MST1 [59]. Very interestingly, MST1 is described in this report as a direct negative regulator of IRF3. MST1 phosphorylates Thr75 and Thr253 on IRF3 to abolish all IRF3-mediated transcriptional responses in vitro and in vivo. Further, virus-induced TBK1-IKKε signaling was disabled by MST1. Evidently, these results are contradictory to the findings of both Wang et al. and Boro et al.. Thus, it is possible that there exists very sensitive, system-specific functions for the Hippo components in immune regulation that depend upon host species, pathogen identity, cell lines used and possible clonal variability.

In addition to VSV, SeV and HSV-1, *Salmonella* may also exploit the Hippo pathway to evade host innate immunity. Perez-Lopez et al. demonstrated that *Salmonella* downregulates YAP activity in B lymphocytes to dampen bactericidal mechanisms [60]. *Salmonella* infection is generally controlled by the detection of intracellular pathogen-associated molecular patterns (PAMPs, e.g., flagellin) by Nod-like receptors (NLR). Detection of PAMPs by NLRC4 in macrophages induces inflammasome assembly and pyroptosis—inflammatory programmed cell death. In B lymphocytes however, *Salmonella* enhances S127 phosphorylation and inactivation of YAP, thereby reducing transcriptional activation of its downstream target NLRC4 and suppressing the initiation of the antimicrobial response.

**Figure 3:**
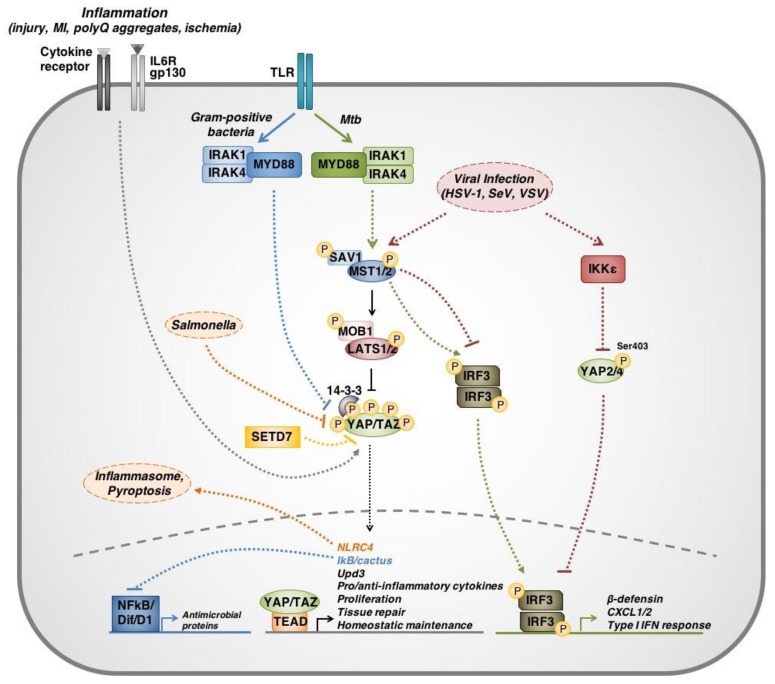
Overview of Hippo signaling immunomodulatory functions. Hippo pathway components participate in a number of both canonical and non-canonical signaling mechanisms that regulate, or are regulated by, immune responses and immune challenges. (1) Through TLR signaling, different stimuli have been shown to trigger different signal transduction cascades. Gram-positive bacteria have been shown to suppress YAP/TAZ transcriptional activity, which upregulates NFkB transcriptional activity through IkB/cactus and expression of antimicrobial proteins. On the other hand, *Mycobcterium tuberculosis (Mtb)* pathogenesis is associated with non-canonical MST1-IRF3 signaling to induce an antipathogenic response, independent of LATS1/2 and YAP/TAZ. (2) Inflammation as a result of insults (myocardial infarction, neuronal polyglutamine aggregates, or ischemia) can upregulate YAP/TAZ signaling through a number of mechanisms that are involved in augmenting tissue repair and mitigating deleterious cytotoxic inflammation. (3) Viral infection (herpes simplex virus (HSV-1), Sendai virus (SeV), vesicular stomatitis virus (VSV)) has been associated with both inducing and suppressing antiviral immunity. This mechanism involves suppression of IRF3 signaling by non-canonical MST1 function to dampen type I interferon response, or phosphorylation at Ser403 and degradation of YAP2/4 to relieve YAP-mediated suppression of antiviral response. (4) *Salmonella* infection has been shown to induce inflammasome assembly through NLRC4 in macrophages. In B cells however, *Salmonella* infection inhibits YAP/TAZ transcriptional activity, therefore reducing expression of Nod-like receptor C4 (NLRC4) and inhibiting pyroptosis, ultimately enhancing *Salmonella* survival within B cells. (5) SETD7 suppresses YAP/TAZ function in the midgut. Parasitic infection by helminths has been shown to reduce SETD7 expression, thereby relieving YAP/TAZ inhibition and accelerating enterocyte/enteroblast proliferation to enhance helminthic clearance. Solid lines represent direct interactions. Dotted lines represent mechanisms that are indirect or have not been fully delineated.

While there is much discrepancy surrounding the precise role and direction of Hippo signaling in anti-pathogenic immunity, this discrepancy might be better regarded as sensitive, system-dependent functionality (Figure 3). Collectively, these results suggest that Hippo plays a crucial role in anti-pathogenic immunity, which is highly dependent on the model used, and the context in which the results are interpreted. It is therefore important that further efforts are invested into mapping the complexities of immune-related Hippo functions.

### 3.2. Hippo Signaling Links Immune Responses with Tissue Regeneration

There is emerging evidence that physiological Hippo signaling is not only important for anti-pathogenic immunity but also might serve to link the activation and resolution phases of an immune response. In the context of tissue injury, Hippo signaling may be differentially regulated by multiple inputs, including contact inhibition, mechanotransduction and inflammatory mediators [61]. In their description of Yki-induced Upd3 expression, Houtz et al. showed that Yki and Scalloped function within a Misshapen (Msn)-Wts-Yorkie/Scalloped-Upd3 signaling axis that enhances intestinal tissue renewal during the *Drosophila* midgut response to *Ecc15* bacterial infection [55]. In mouse models, Nowell et al. demonstrated that chronic inflammation of the corneal epithelium changes the composition of the extracellular matrix leading to activation of mechanotransduction, nuclear translocation of YAP/TAZ, increased β-catenin signaling and metaplasia [62]. Finally, YAP/TAZ have been described in enhancing cardiac functional recovery following MI [42]. It has been proposed that inflammation-induced Hippo signaling may be critical for repairing tissue damage sustained during a mucosal injury/pathogen infection. Tanaguchi and coauthors showed that the IL-6 co-receptor, gp130, activates YAP through SRC-family kinases and that activated YAP is important for intestinal regeneration after dextran sulfate sodium salt challenge [63]. Indeed, while each of these studies implicates the Hippo pathway effectors, YAP/TAZ/Yki, in tissue recovery after inflammation, the precise mechanisms leading to activation of the effectors differs in each model. It is possible that multiple, converging pathways activate YAP/TAZ/Yki in damaged tissue and that the physical/biological properties of a wound determine the pathway through which the Hippo effectors are activated.

While the roles of Hippo signaling in nervous system development and pathophysiology remain unclear, novel findings have linked YAP to neuroinflammatory processes. Specifically, Yki has been implicated in mitigating the deleterious inflammation associated with innate immune activation by polyglutamine (PolyQ) aggregates within neurons, suggesting an anti-inflammatory neuron-intrinsic function for Yki [64]. In fact, YAP has been reported to suppress inflammatory astrogliosis by transcriptionally upregulating SOCS3 from within astrocytes, ultimately leading to inhibition of STAT3 and STAT3-mediated inflammation [65]. With inflammation being an important component of most neurodegenerative processes, these studies offer important preliminary data for future directions.

It should also be noted that the relationship between inflammation, the Hippo pathway and tissue regeneration has important applications in cancer biology. Indeed, enhanced YAP activity within inflamed tissues may contribute to tumourigenesis. For example, Tanaguchi et al. have proposed that *APC*-mutated colon cancers have greater expression of gp130 and increased sensitivity to local IL-6, IL-11 or sIL-6R [66]. They further show that increased gp130 signaling in these colon cancer cells sustains YAP activation and YAP subsequently upregulates gp130 expression through TEAD4 as part of an autoregulatory feedback loop. Thus, an understanding of how the Hippo pathway is affected by inflammation in general may have specific relevance in a neoplastic context.

## 4. Immune Cell-Intrinsic Hippo Signaling

### 4.1. Clinical Evidence for Immune Cell-Intrinsic Hippo Signaling

**Figure 4:**
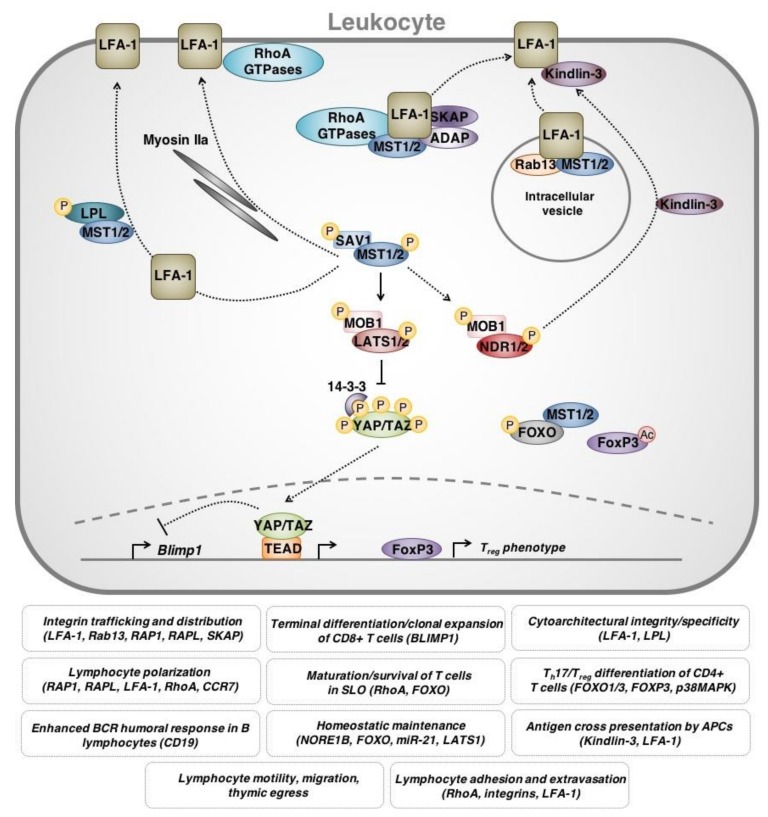
Overview of leukocyte-intrinsic Hippo pathway functions. Hippo pathway components are involved in regulating leukocyte activity, namely T lymphocytes. MST1 is also involved in both canonical and non-canonical signaling involved in T lymphocyte proliferation and survival. (1) MST1 plays a crucial role in regulating the cytoarchitecture of T lymphocytes to facilitate polarization, formation of the leading edge, adhesion and migration. This function is thought to be dependent upon the regulation of the integrin leukocyte function-associated antigen-1 (LFA-1). Direct and indirect interactions between MST1 and other proteins, including endosomal adaptor protein Rab13, cytoskeletal regulatory protein Kindlin-3, a number of RhoA GTPases (RAP-RAPL complex, Rac1), L-plastin (LPL) and myosin IIa all lead to proper LFA-1 activation, spatiotemporal distribution and appropriate low/high affinity partitioning on T lymphocyte membrane. These interactions allow for T lymphocyte migration, survival and adhesion, as well as enable correct thymic egress and secondary lymphoid organ infiltration. (2) MST1/2 signaling is also involved in CD4+ T lymphocyte T_h_17 differentiation, through interactions with FOXO1/3 and upregulation of FoxP3 transcriptional activity. (3) Hippo signaling is also involved in regulating clonal expansion of CD8+ T lymphocytes vs. terminal differentiation, through transcriptional regulation of Blimp1. (4) Hippo signaling through LATS1 or MST/FOXO interactions is implicated in apoptotic control, homeostatic maintenance of T lymphocytes and control of oxidative stress. Solid lines represent direct interactions. Dotted lines represent mechanisms that are indirect or that have not been fully delineated.

Some compelling evidence for the role of Hippo signaling in the immune system comes from clinical case reports of patients who have disrupted MST1 expression/function. Inherited mutations in the gene that encodes MST1 (*STK4*) have been described and are associated with immune phenotypes. Specifically, MST1 loss causes combined immunodeficiency with increased susceptibility to bacterial/viral/fungal infections as well as autoimmune signs/symptoms (e.g., hypergammaglobulinemia and autoantibody production) [67,68,69,70,71]. Hypermethylation of the *MST1/STK4* promoter and reduced MST1 expression has also been noted in patients with autoimmune pancreatitis (with extrapancreatic lesions) and rheumatoid arthritis [72]. Interestingly, studies examining the molecular mechanisms underlying these associations have demonstrated that MST1 loss within leukocytes is primarily responsible for the clinical presentation. Indeed, patients with MST1 dysfunction are lymphocytopenic and show profound deficits in T cell maturation, trafficking, responsiveness and viability [67,68,69,71]. Abnormalities in neutrophil viability have also been reported [69]. These pathologies are accurately recapitulated in *Mst1/Stk4*-knockout mouse models [73,74,75,76]. *Mst1/Stk4*-knockout mouse models have also been used to implicate MST1 in experimental autoimmune encephalomyelitis and collagen-induced arthritis [75]. Therefore, it is clear that MST1 plays vital roles in normal immune cell function, immune homeostasis and immune-related disease (Figure 4) [77,78].

### 4.2. MST1 in the Motility and Trafficking of T Cells

The hierarchy of leukocytes begins at the level of haematopoetic stem cells (HSC), which generate both common lymphoid progenitor (CLP) and common myeloid progenitor (CMP) cells within the bone marrow. CLPs give rise to T cell progenitors, which develop and mature within the thymus. In the thymus, T cells undergo selection to eliminate T cell clones with T cell receptors (TCR) that bind to self major histocompatibility complex (MHC)-peptide complexes with too high or too low affinity. Through a number of developmental stages, mature T cells that are either CD4+ or CD8+ emerge from the thymus to browse antigens within peripheral tissue and secondary lymphoid organs. B cell progenitors also arise from CLPs, and these continue to mature and develop into B cells within the bone marrow and secondary lymphoid tissue such as the spleen. Over the past two decades, there has been substantial growth in the body of literature examining the role of Hippo components in the development and maturation of lymphocytes. Interestingly, MST1 is abundantly expressed in lymphoid tissue, where it affects the development, maturation, functionality and trafficking of T lymphocytes through both primary and secondary lymphoid tissue.

Numerous studies have established the critical functions of MST1 in the thymic egress of CD4+/CD8− and CD4−/CD8+ single-positive T cells. Indeed, targeted disruption of MST1 results in significantly reduced levels of peripheral T cells and impaired T cell functions pertaining to adhesion, homing and interstitial motility [74,79,80,81]. MST1 kinase activity is required for T cell polarization following TCR stimulation in human and murine in vitro models [73,74,79]. Mou et al. (2012) reproduced similar findings in vivo, demonstrating that *Mst1/2* DKO mice exhibited reduced levels of mature T lymphocytes in circulation and within secondary lymphoid tissues [82]. Single-positive MST1/2-deficient T lymphocytes were unable to enter secondary lymphoid tissues, exhibited accelerated apoptosis, and had severely diminished thymic egress and motility. Mechanistically, MST1 is thought to primarily function within leukocytes through non-canonical interactions. In fact, whether MST1/2 exist within a Hippo signaling network and interact with LATS1/2, YAP and TAZ within immune cells is unclear [82]. Katagiri and colleagues have determined that MST1/RAPL complexes are essential for transporting the integrin LFA-1 to the T lymphocyte leading edge during polarization and adhesion [79,80]. In vitro, MST1 deficient lymphocytes exhibit further defective trafficking of α4-integrins and significantly diminished adhesion. *Mst1/2* DKO also impaired Rac1 and RhoA GTP charging, thereby inhibiting T lymphocyte migration and adhesion [82]. Interestingly, *Ndr1/2* kinase DKO mice phenocopy *Mst1/2* DKO mice, suggesting that NDR kinases may act downstream of MST1/2 in T lymphocyte regulation as opposed to LATS1/2 [83].

Other studies have reinforced the importance of MST1 function in CD4+ T lymphocyte thymic egress/antigen recognition and the requirement for integrins (e.g., LFA-1) in this process. Indeed, MST1 appears to be central for regulating LFA-1 localization and activation through “inside-out signaling” [81]. This cascade begins with TCR or cytokine receptor activation and is mediated through a number of intracellular protein complexes to ultimately localize LFA-1 to the leading edge of polarized T lymphocytes [84,85,86]. The chemokine receptor CCR7 has been identified as an initiator of this cascade, and acts through the ADAP/SKAP55 complex and MST1 to augment LFA-1 localization and activation [87]. Within murine lymph nodes, Raab et al. (2010) showed that SKAP interacts with Rap1-RAPL complexes and requires MST1 for appropriate LFA-1 trafficking and subsequent T cell interactions with DCs [85]. Further studies found that MST1 regulates Myosin IIa localization, enabling actin-dependent distribution and partitioning of LFA-1 along the membrane of migratory T lymphocytes [88]. MST1 was moreover found to necessarily associate with and phosphorylate the actin-binding protein, L-plastin, which is a crucial interaction for T cell polarization and migration [89]. In fact, L-plastin-deficient mice phenocopy MST1-deficient mice. Additionally, Rab13 endosomal adaptor protein has been recently found to associate with MST1 [90]. The Rab13-MST1 complex colocalizes with LFA-1 within intracellular vesicles to enable proper LFA-1 trafficking to the leading edge of polarized T lymphocytes. Thus, Rab13 deficient mice exhibit leukopenic lymphoid tissue that is attributable to defective LFA-1 trafficking and restricted T lymphocyte adhesion and motility. These findings underline a novel role for the MST1 kinase in in endosomal kinetics and spatiotemporal regulation within T lymphocytes.

### 4.3. Hippo Signaling in the Function/Differentiation of T Lymphocytes

MST1 has also been reported to play a role in determining T cell proliferation and survival. Using both mouse in vitro and in vivo studies, Zhou and colleagues demonstrated that MST1, and its regulatory protein NORE1B, suppress the proliferation of naïve mature CD8+ T lymphocytes in a LATS independent manner [73]. However, genetic ablation of murine *Mst1* has also been determined to reduce T cell proliferation and IL-2 production while enhancing apoptosis, in vitro, and, consistent with this, it has been suggested that YAP negatively regulates prolifration of CD4+ T lymphocytes, contrary to its pro-proliferative function in epithelial cells [75,91]. In either case, it seems that MST1 influences the proliferative rate of T cells either through conventional Hippo siganling or through Hippo-independent interactions. With respect to cell survival, MST1 also acts as a regulator of oxidative stress-induced apoptosis in peripheral naïve T cells through interaction with the FOXO signaling pathway [92]. MST1-deficient mice demonstrated higher levels of intracellular ROS, lower levels of SOD2 and catalase as well as higher rates of apoptosis. Downstream of MST1, LATS1 was identified as a novel target of antiapoptotic microRNA-21 in Jurkat T cells [93]. Hippo signaling has also been implicated in coordinating proliferation during clonal expansion and terminal murine CD8+ T lymphocytes [94]. Viral infection prompts YAP-induced proliferation within T cell clones and physical interactions between expanded T cells leads to contact-inhibition and Hippo-mediated YAP degradation. This leads to enhanced expression of Blimp1 and terminal differentiation of CD8+ T cells.

MST1 has also been reported to maintain peripheral tolerance to prevent autoimmune reactions in the context of pathogen infection. Interestingly, MST1 expression in DCs plays a role in pro-inflammatory T_h_17 differentiation and antifungal immunity. Li et al. show that fungal infection induces signaling through p38MAPK within DCs and this is antagonized by MST1 [95]. In the absence of MST1, the p38MAPK cascade ultimately results in IL-6R stimulation and STAT3 activation within CD4+ T lymphocytes and adoption of a T_h_17 phenotype. In addition to this, MST1 suppresses inflammation through regulatory T cells [96]. In vitro and in vivo mouse studies have revealed compelling evidence that MST1 enhances the expression of Foxp3, a master regulator for T_reg_ development, thereby enhancing T_reg_ function and anti-inflammatory responses. Mechanistically, MST1 directly phosphorylates and stabilizes Foxo1 and Foxo3, dampens TCR-induced AKT activation and ultimately enhances Foxp3 expression. MST1 was additionally found to augment Foxp3 protein stability through modulating Foxp3 acetylation [97]. Thus, through both kinase-dependent and -independent mechanisms, MST1 maintains immune tolerance through T_reg_ modulation. Collectively, these findings shed light onto the convoluted mechanisms precipitating autoimmunity and immune-deficiency in human patients with aberrant *MST1*. These data also collectively support the role of Hippo in the survival and homeostatic maintenance of naïve T cells and self-tolerance.

### 4.4. Hippo Signaling in the Immunological Synapse

In addition to regulating T cell maturation and development, MST is important for T cell activation by antigen presenting cells (APCs). MST has been implicated in the formation of the immunological synapse—the interface between lymphocytes and APCs/target cells where TCR and peptide-MHC complexes interact. Tomiyama et al. found that MST1-knockout T_reg_ have defective immunological synapse formation and impaired interactions with DCs [98]. Further, Kondo and coauthors later showed that MST1 signals through NDR1 during supramolecular activation cluster (SMAC) maturation to regulate the localization of kindlin-3 in naïve T cells [99]. Kindlin-3 stabilizes the immunological synapse, allowing high-affinity binding between LFA-1 (on T cells) and ICAM-1 (on APCs). MST1 also appears to be critical for immunological synapses involving B cells albeit through a different mechanism. MST1 positively regulates the B cell receptor (BCR) stimulatory co-receptor CD19 presumably through canonical Hippo signaling and TEAD2 [100]. MST1-deficient mice therefore show reduced CD19 expression, disrupted BCR clustering/downstream signaling and poor marginal zone B cell viability. Consistent with this, Salojin et al. (2014) reported that MST1-deficient B lymphocytes were markedly unresponsive to mitogenic stimulation of the BCR in vitro and that MST1-deficient mice did not produce a significant humoral response to ovalbumin [75]. More recent evidence by Park et al. suggests a more complex in vivo regulatory network, whereby MST1 mediates cross-talk between T_regs_, T_h_2, and B lymphocytes [76]. In this model, MST1 deficient mice exhibited a hyperactivated B lymphocyte-mediated humoral response as a result of defective T_reg_ immunomodulatory signaling, indicating multidirectional regulation between lymphocyte subtypes modulated by cell-intrinsic MST1 functions.

### 4.5. Hippo Signaling in Other Leukocytes

While much of the literature surrounding leukocyte-intrinsic Hippo function has focused on lymphoid cells, there is evidence that MST is also important for immune responses involving other cell types. Indeed, disrupted T cell function makes up only one aspect of the phenotype observed in *Mst1*-knockout mice. Katagiri observed that MST1-deficient mice exhibit hypotrophic peripheral lymphoid tissue as well as impaired B cell and DC maturation in the spleen [80]. In addition to regulating T cell function, CCR7 was also found to alter human mature DC endocytosis, migration, and cytoarchitecture by acting through the RhoA pathway and kinase-dependent MST1 functions [101]. Neutrophils also exhibit similar phenotypes to T lymphocytes in *Mst1*−/− mice, including impaired migration, adhesion/extravasation and response to immune challenge likely due to defective intracellular trafficking of crucial neutrophil integrins, including VLA-3 and VLA-6 [102]. To test the contributions of MST1/2 to myeloid immune cell function, Geng et al. generated a mouse model with myeloid cell-specific *Mst1/2* knockout and showed that MST1/2 are essential components of the phagocytic response to bacterial infection [103]. Mechanistically, in the context of bacterial infection, TLR signaling activates MST1/2 which further leads to activation of the GTPase Rac, assembly of a TRAF6-ECSIT complex and mitochondrial trafficking/juxtaposition with the phagosome. This phagocyte MST1/2 activity may also have relevance to non-infectious disease processes involving macrophages (e.g., atherosclerosis) or microglia (e.g., ischemia-reperfusion injury) [44,104].

### 4.6. Leukocyte-Intrinsic Hippo Signaling in Cancer

Immune cell-intrinsic Hippo signaling should not be overlooked in the context of cancer. There is evidence that MST1 can alter anti-neoplastic immune responses from within lymphocytes. CTLs with MST1-deficiency show reduced expression of FoxO1 and FoxO3a (negative regulators of CD8+ T cell function) [105]. While MST1 loss and diminished FoxO1/3 levels may increase naïve T cell susceptibility to cellular stress and apoptosis, Yasuda et al. have found that these cells also have higher levels of T-bet, IFNγ and granzyme B [92,105]. Functionally, these *Mst1*-knockout CTLs display enhanced cytotoxicity against tumour cells in vitro and greater suppression of tumour growth in vivo in a mouse thymoma (EG7-OVA) tumour model. YAP overexpression within tumour-associated CD4+ T lymphocytes has been suggested to induce differentiation into T_reg_ cells [106]. The T_reg_-induced immunosuppressive tumour microenvironment contributes towards cancer immune evasion and correlates with poor prognosis in hepatocellular carcinomas. In contrast, TAZ expression in CD4+ T lymphocytes attenuates T_reg_ differentiation and favours T_h_17 differentiation [107]. In accordance with these findings, Buglioni et al. (2016) have shown that the prognostic value of YAP/TAZ expression within cervical cancer cells becomes confounded by the YAP/TAZ expression levels of tumour-infiltrating lymphocytes (TILs) [108]. While elevated YAP/TAZ levels within cancer cells generally correlated with poor prognosis, elevated levels of YAP/TAZ within TILs correlated with enhanced responsiveness to neoadjuvant chemotherapy, possibly due to enhanced clonal expansion and effectiveness of anti-neoplastic CD8+ T cells. Thus, there are mechanisms by which immune cell-intrinsic Hippo signaling can augment the anti-neoplastic immune response or can participate in cancer immune evasion from a different perspective.

Given this, it is clear that careful consideration of the functions of Hippo signaling within cancer cells and also within immune cells will be necessary in order to accurately interpret in vivo experimental data or when contemplating whether Hippo pathway core components might be practical therapeutic targets for treating cancer.

## 5. Conclusions and Future Directions

The Hippo signaling pathway, initially described in *Drosophila* and later delineated in mammalian systems, is fundamental in organ size control and organism development. While the Hippo pathway has gained considerable interest due to its roles in human cancers, it has also been implicated in regulating host immune responses. Such findings suggest that the Hippo pathway not only promotes cancer initiation and progression through proliferative cancer cell-autonomous effects, but also by facilitating the establishment of an appropriate immunosuppressed tumour microenvironment.

Given the diverse roles of Hippo signaling throughout immunology, further validation of immune-related transcriptional targets of YAP/TAZ represents a major area for future work. Transcriptional regulation of genes like PD-L1, CXCL5, CCL2 and CSF1 are important mechanisms by which YAP/TAZ augment immune cell activity. Previous searches for YAP/TAZ-regulated genes revealed many other candidate gene targets that are relevant to immunology [21,109,110,111,112,113]. For example, cytokines including IL1α/β, IL8, CXCL1/2/3 were among the top genes that we characterized as being downregulated by TAZ overexpression in MCF10A immortalized breast epithelial cells [114]. Similarly, in our NanoString-based screen, we identified many candidate immune-related YAP/TAZ targets including cytokines and their receptors (e.g., CXCR4, CCL2), complement factors (e.g., CFI, C3) as well as components of pattern-recognition receptors (e.g., NLRP3, CD14) [45]. Functional validation of these and other immune-related genes as bona fide transcriptional targets of YAP/TAZ may provide new insights into physiological and pathological functions of Hippo signaling. Likewise, it may be worthwhile for future studies to revisit supplementary data from the existing literature to identify other interactions warranting further exploration.

Signal transduction is rarely conceptualized as a linear mechanism, rather it should be approximated as a network of multidirectional interactions that are highly dependent upon the context in which they are investigated. It is for this reason that the growing body of literature presents conflicting data, which remain to be reconciled. Future studies should aim to evaluate Hippo pathway function across species and cell types to ensure that key observations are generalizable. Finally, it may be worthwhile for future studies to reexamine the contribution of the Hippo pathway effectors and canonical signaling to immune cell development. This is especially timely given the recent literature about the role of YAP/TAZ in T cell differentiation. While we have made ample progress in our understanding of Hippo and the immune system, our efforts must continue if we aim to shape a holistic and comprehensive signaling model.
